# A community-based parent-support programme to prevent child maltreatment: Protocol for a randomised controlled trial

**DOI:** 10.12688/hrbopenres.12812.2

**Published:** 2018-09-21

**Authors:** Mairead Furlong, Ann Stokes, Sinead McGilloway, Grainne Hickey, Yvonne Leckey, Tracey Bywater, Ciaran O’Neill, Chris Cardwell, Brian Taylor, Michael Donnelly, ENRICH Research Team

**Affiliations:** 1 Centre for Mental Health and Community Research, Department of Psychology, John Hume Building, National University of Ireland Maynooth, Maynooth, Co. Kildare, Ireland; 2 University of York, York, UK; 3 Queens University Belfast, Belfast, UK; 4 Ulster University, Derry, UK

**Keywords:** Child maltreatment, child abuse and neglect, family, parenting, wraparound

## Abstract

The prevention of child abuse and neglect is a global public health priority due to its serious, long-lasting effects on personal, social, and economic outcomes. The Children At Risk Model (ChARM) is a wraparound-inspired intervention that coordinates evidence-based parenting- and home-visiting programmes, along with community-based supports, in order to address the multiple and complex needs of families at risk of child abuse or neglect. This paper presents the protocol for a study that will be carried out to evaluate this new service model (i.e. no results available as yet). The study comprises a multi-centre, randomised controlled trial, with embedded economic and process evaluations. The study will be conducted in two child-welfare agencies within socially disadvantaged settings in Ireland. Families with children aged 3-11 years who are at risk of maltreatment (n = 50) will be randomised to either the 20-week ChARM programme (n = 25) or to standard care (n = 25) using a 1:1 allocation ratio. The primary outcomes are incidences of child maltreatment and child behaviour and wellbeing. Secondary outcomes include quality of parent-child relationships, parental stress, mental health, substance use, recorded incidences of substantiated abuse, and out-of-home placements. Assessments will take place at pre-intervention, and at 6- and 12-month follow-up periods. The study is the first evaluation of a wraparound-inspired intervention, incorporating evidence-based programmes, designed to prevent child abuse and neglect within high risk families where children are still living in the home. The findings will offer a unique contribution to the development, implementation and evaluation of effective interventions in the prevention of child abuse and neglect.

The trial is registered with the International Standard Randomised Controlled Trial Number Register (DOI 10.1186/ISRCTN13644600, Date of registration: 3 ^rd^ June 2015).

## Introduction

It is widely acknowledged that child abuse and neglect (also called child maltreatment [CM]) has serious and far-reaching consequences, contributing to a range of negative outcomes. These include: child mortality and morbidity; long-term effects on mental health; drug and alcohol misuse; risky sexual behaviour; poor educational and employment prospects; criminality; lower life expectancy; intergenerational transmission of maltreatment; and increased expenditure on health, judicial and social welfare services ( Gilbert *et al*., 2009; Sethi *et al.*, 2013). Thus, the prevention of child abuse and neglect is an important human rights and global public health priority. Recent meta-analyses of self-reported incidences of CM have indicated that emotional abuse is the most common type of CM (36.3%), followed by physical abuse (22.6%), neglect (16.3% physical and 18.4% emotional) and sexual abuse (18.0% [girls] and 7.6% [boys]) ( Stoltenborgh *et al.*, 2012; Stoltenborgh *et al.*, 2013a). However, it is possible that some or all of these are underestimates because prevalence rates can vary according to methodological factors ( Stoltenborgh *et al.*, 2013a; Stoltenborgh *et al.*, 2013b).

Furthermore, due to unreliable detection and surveillance systems in most countries (including Ireland), official statistics of substantiated abuse are widely believed to seriously underestimate the occurrence of CM, with reports suggesting that 90 per cent of child abuse and neglect goes unnoticed ( Munro, 2011; Stoltenborgh *et al.*, 2013a; Stoltenborgh *et al.*, 2013b). Self-reports are considered more accurate, but are still likely to underestimate true prevalence rates ( Gilbert *et al.*, 2009). Incidences of substantiated abuse vary between countries, but studies indicate that children of all ages (but especially those who are younger) are at risk of abuse and neglect ( Akmatov, 2011). For instance, in the US in 2013, children under three years had a CM rate of 14.3 per 1000, compared with 10.3 per 1000 for children ages four to seven, 7.6 for children ages eight to 11, 6.7 for children ages 12 to 15, and 4.5 per 1000 for children ages 16 to 17 ( U.S. Department of Health and Human Services, Administration on Children, Youth and Families, 2013). In Ireland, over 40,000 referrals of child welfare and abuse cases were made to social work annually during 2012–2014, which represents a rate of 35 per 1000 children; this was almost double the number referred in 2007 ( Tusla Quarterly National Performance Activity Report, 2015). These figures (although unlikely to be all confirmed cases) are a source of considerable concern and may be related, at least in part, to the impact of the economic recession in Ireland, including unemployment, financial difficulties and homelessness, all of which have been a feature of life in Ireland in recent years ( Williams *et al.*, 2016).

Importantly, despite a ratio of investment of 90 to 1 in child protection versus prevention services in the US and Europe ( Gilbert *et al.*, 2009), attempts to treat the consequences of CM are less effective, more costly, and ethically inferior to investing in programmes to prevent CM and family breakdown ( Leventhal, 2005). Furthermore, prevalence rates of CM are even higher in low and middle-income countries than in high-income countries, thereby making CM a truly global phenomenon ( Sethi *et al.*, 2013).

Child maltreatment may be explained by multi-systemic factors. The most significant risk factors for child abuse and neglect may be best understood within an ecological risk framework ( MacKenzie *et al.*, 2011); these relate to poor parenting behaviours and parental stress, parental mental illness, parental experience of being maltreated as a child, parental substance abuse, family conflict, child misbehaviour and disability, and social disadvantage (e.g. young, single parents with low education and income levels) ( Stith *et al.*, 2009). Research on protective factors to prevent CM is less developed than studies which have focused on identifying and understanding risk factors ( Sethi *et al.*, 2013). ‘Protective factors are defined as characteristics within the individual, family, or community associated with a lower likelihood of problematic outcomes’ ( National Research Council and Institute of Medicine Committee on the Prevention of Mental Disorders and Substance Abuse Among Children, *et al.*, 2009: p.82). Despite wide variation in study designs and samples, family-level protective factors, such as a stable family environment and supportive relationships, show a consistent association with resilience following child maltreatment. There is also evidence for some individual-level factors, such as personality traits. Community protective factors include, amongst others, peer relationships, non-family member relationships, non-family member social support, and religion ( Afifi & MacMillan, 2011). Therefore, there is increasing international recognition of the need to coordinate services and supports in order to address the complex needs of vulnerable families at risk of CM, who are often involved in multiple, ‘siloed’ systems of care ( Burns *et al.*, 2000; Sethi *et al.*, 2013).

### Interventions to address CM

This protocol relates to a study which involves the evaluation of a ‘wraparound inspired’ early intervention and prevention programme - called ChARM (Children At Risk Model) - which provides comprehensive parenting and family supports aimed at addressing CM and improving child wellbeing within high risk families whose children are aged 3–11 years. The ChARM service model incorporates evidence-based intervention and prevention programmes (i.e. home visiting and the Incredible Years BASIC group-based parenting programme), as well as a newly developed positive life-skills programme and other community-based supports which are provided as necessary to address specific family needs. This study is the first evaluation of a ‘wraparound-inspired’ approach, incorporating evidence-based programmes to tackle child maltreatment within high risk families where children are still living in the home. 

The wraparound (WA) model of care which inspired the development of the ChARM intervention, was developed in the US in the 1980s. It offers a family-focussed and strengths-based intervention approach which involves coordinating available formal and informal supports to meet the multiple needs of families. WA has demonstrated effectiveness in improving placement stability and psychosocial functioning among youths with serious mental health and behavioural disorders ( Suter & Bruns, 2008; Suter & Bruns, 2009). WA individualizes a combination of services selected to be "wrapped around" families in contrast to stand-alone, standardized intervention approaches ( Winters & Metz, 2009). Due to its individualized nature, the effectiveness of WA programmes is influenced by the ‘fit’ between family needs and the quality of services available within the local community system ( Bruns *et al.*, 2008). WA is not based on any single theory of change; instead, it is consistent with several influential psychosocial theories of child development and behaviour, including the social-ecological approach, social learning theory, and systems theory ( Walter & Petr, 2011).

Preliminary evidence from a retrospective cohort study indicated that both families with children still living in the home and foster care families who received the Brevard C.A.R.E.S (Coordination, Advocacy, Resources, Education and Support) wraparound intervention had reduced incidences of verified maltreatment compared to usual services ( Schneider-Muñoz *et al.*, 2015). By contrast, a randomised controlled trial of WA versus standard services for maltreated children within those still living in the home and in out-of-home placements reported no differences in child and carer wellbeing ( Browne *et al.*, 2014). It has been noted that, while WA improves placement stability and is perceived as being a highly transportable and acceptable approach to working with families within current care systems, it tends to have less support than evidence-based programmes (EBPs) in improving clinical outcomes ( Bernstein *et al.*, 2015). Conversely, EBPs may lack feasibility and generalizability ( Bruns *et al.*, 2014). There is increasing recognition, therefore, that a WA approach, or indeed an approach inspired by wraparound principles, that incorporates evidence-based CM prevention programmes, while also coordinating other tailored community-based supports, may offer a useful model of care in enhancing both clinical outcomes and programme feasibility ( Bernstein *et al.*, 2015).

Evidence from meta-reviews has indicated that, of available EBPs, home visiting and group-based parent training programmes appear most successful in improving risk factors associated with CM, and to a far lesser extent, in reducing incidences of CM ( MacMillan & Wathen, 2014; Mikton & Butchart, 2009). Nevertheless, there is little evidence to suggest that these stand-alone parenting programmes are sufficient in preventing CM in more high risk, disadvantaged families. For example, many ‘real world’ implementation studies have shown that less than 30–50 per cent of vulnerable families will attend a centre-based parenting programme and that more than half of these will drop out during delivery ( Axford *et al.*, 2012; Furlong & McGilloway, 2015). Such failure to engage parents is unsurprising because, arguably, stand-alone parent programmes are typically not equipped to address the multiple and complex needs of families at risk of CM, which as outlined earlier, include addiction and mental health problems, housing and financial concerns, and so forth. Home-visiting interventions, on the other hand, appear to have more capacity than parenting programmes to engage with vulnerable families due to meeting within the family home and addressing other material and support needs besides coaching of parenting skills ( Macdonald *et al*., 2010).

Nevertheless, reviews report mixed results, particularly if home visitors have heavy caseloads, do not adopt a collaborative approach, and fail to coordinate the provision of necessary supports (e.g. mental health and addiction services) ( Gomby, 2005). Additionally, a meta-review indicated that there is little evidence that stand-alone home visiting is effective in reducing incidences of CM (it is more successful in addressing risk factors for CM) ( Mikton & Butchart, 2009). Moreover, it should be noted that, to date, most evaluations of preventive home-visiting programmes target families with very young children (0–3 years) and, therefore, there is a lack of evidence for their effectiveness in reducing CM among families with children older than three years ( Selph *et al.*, 2013). The lack of evidence for home-visiting interventions targeted at older children is unexpected in light of: (1) reports that indicate that CM may remain undetected for years and only manifest at a later age ( Sethi *et al.*, 2013); (2) substantiated and self-reports that indicate a high occurrence of CM in children aged between three and 11 years ( Stoltenborgh *et al.*, 2013a; Stoltenborgh *et al.*, 2013b); and (3) the availability of home-visiting supports in many countries for families where the child is older than three years ( Children and Young People Now Jobs, 2017; Tusla, 2017).

Arguably, therefore, home visiting and parenting programmes are not sufficient, when delivered as stand-alone interventions, to meet the complex needs of vulnerable families. Preliminary evidence from meta-analyses of parenting supports to prevent child abuse has indicated that interventions which combine home-visiting elements and group-based parent training may be more effective in improving risk factors associated with CM than either component delivered on its own ( Chen & Chan, 2016; Lundahl *et al.*, 2006). Therefore, despite their limitations as stand-alone interventions in engaging high-risk families, it may be advisable to incorporate evidence-based home visiting and parenting programmes within a WA intervention. A WA-inspired approach that coordinates home visiting and parent training with other tailored formal and informal supports may also address family needs not otherwise met, such as parental and child mental health, substance misuse, domestic abuse, resilience and social skills competencies, and housing and financial difficulties. If found to be effective in preventing risk factors and incidences of CM, an intervention inspired by WA principles such as the ChARM model which is the subject of this study, may achieve considerable cost savings in terms of reduced utilization of child welfare services, foster and residential home placements, criminal justice, mental health, prison service and other long-run costs that are typically incurred when children are exposed to abuse and neglect ( Corso & Lutzker, 2006).

### The Irish context

Child welfare and protection policy in Ireland is based on a legal framework provided primarily by the Child Care Act 1991 and the Children First Act 2015. Tusla ( The National Child and Family Agency) has a statutory responsibility to assess all reports of child welfare and protection concerns in Ireland. Assessments are carried out by Tusla social workers. If concerns are found after the initial checks, a further assessment is carried out, involving a detailed examination of the child and family’s circumstances. If concerns about a child’s welfare are found, but do not involve a child protection issue, then the family may be referred to community or family support services. If no concerns are found, then the information gathered is recorded and kept on a confidential file where it will be examined if further concerns or more information comes to light ( Children First: National Guidelines, 2017).

The development and implementation of a WA model of care for child and family services in Ireland is currently undergoing a period of transition and is at a different stage of advancement to WA as established in the National Wraparound Initiative (NWI) in the US ( NWI, 2017). In recent years, a number of policy initiatives in Ireland have emphasized the importance of interagency collaboration and service coordination in order to improve outcomes for children and families ( Better Outcomes Brighter Futures, 2014; Tusla, 2015). Stand-alone interventions, such as group-based parent training, have struggled to engage more vulnerable families ( McGilloway *et al.*, 2012). Therefore, child welfare organizations have been inspired by a ‘wraparound’ model of care that would coordinate a number of tailored supports to meet the multiple needs of families.

For example, Meitheal is a recent ‘wraparound-inspired’ national practice model that has involved considerable restructuring of services for children in Ireland since 2014; Meitheal is an Irish word that equates to the concept of ‘team around the child’ ( Tusla, 2015). Meitheal is a nine-step model designed to identify child and family needs and strengths and brings together a team around the family to deliver support that is outcomes-focussed, planned, documented and reviewed over time. The support is planned in a highly participatory manner and directed by the family ( Tusla, 2015). As such, Meitheal is similar to the NWI model of care in implementing the ten core wraparound principles. The implementation of Meitheal is also influenced by the Common Assessment Framework in England and Wales, and by the My World Triangle and National Practice Model as part of Getting it Right for Every Child in Scotland ( Tusla, 2015).

While significant progress was made in the implementation of Meitheal within Ireland during 2016 ( Cassidy *et al.*, 2016), it has not yet been sufficiently embedded to have allowed time to restructure the current intervention within its wraparound framework. Therefore, the ChARM model which will be evaluated in this study was developed at an earlier stage (2012 to 2014) than Meitheal and does not contain all WA elements as indicated in the NWI. While it is similar to the NWI wraparound model in terms of utilizing a family-focussed, multi-disciplinary, tailored approach to meet the multiple needs of families, it is different in two important ways. Firstly, there is less flexibility and choice in the current model, as it comprises core components of home visits, parent training and a positive life skills programme (as well as any other supports desired by families). Therefore, the model is targeted towards those families whose needs are best met by such programmes and who agree to engage with them. The US (and Meitheal) model, on the other hand, does not require any mandatory component and allows the family to select any service provider on their team. Secondly, the current model does not involve formal team meetings in which the family and selected service providers are present; rather the family collaborates with a caseworker to produce a coordinated plan of care that is tailored to meet family needs. The plan will include the core components as well as any other requested supports, although access to the latter may depend on availability. Therefore, the current intervention involves an intensive package of supports for families that has been inspired by a wraparound philosophy of care but is not identical to it.

Given the ongoing national implementation of Meitheal, we believe that the ChARM intervention, if shown to be effective, can operate within its framework. Moreover, the current evaluation should help to shed light on whether or not a package of comprehensive community-based supports can prevent child abuse and neglect in high-risk families. For instance, one of the key concerns in establishing Meitheal is that it has developed a WA model of care, but there is a lack of evidence with regard to the types of supports that are most suitable in addressing particular family needs, and the resources and processes required to implement, embed and sustain such supports ( Cassidy *et al.*, 2016).

### The current study

The objectives of the study which is the subject of this protocol, are to evaluate the effectiveness, cost effectiveness and process mechanisms of the ChARM programme for vulnerable families whose children (age 3–11 years) are at risk of maltreatment, when compared to standard services. The primary hypotheses underpinning this randomised controlled trial (RCT) are: (1) the ChARM programme will reduce parent-reported incidences of child maltreatment; and (2) will improve child wellbeing and behaviour. Secondary hypotheses are that the ChARM programme will improve the quality of the parent-child relationship and parenting competencies, reduce parental stress and mental ill health as well as parental alcohol and drug use, and lead to a decrease in recorded incidences of substantiated abuse and out-of-home placements. The embedded process evaluation will investigate programme acceptability and engagement, enablers and barriers to implementation, and mechanisms of impact, while the costs analyses will explore whether the intervention warrants investment compared to standard services. The protocol has followed the SPIRIT guidelines for reporting protocols of clinical trials ( Chan *et al.*, 2013).

## Methods

### Participants

The ChARM programme will be delivered within a social work department and a family resource centre in socio-economically deprived disadvantaged areas of Dublin and Co. Kildare, Ireland. These areas are designated as disadvantaged according to information on demographic profile, academic performance, social class composition, and labour market situation ( Haase *et al.*, 2014).

***Inclusion criteria***

Participants are parents/caregivers of children aged 3–11 years where the child has:

- Been identified by a child welfare professional (social worker, family resource worker) as being at risk of abuse/neglect; or
- Where it is known by child welfare professionals that a level of child maltreatment has occurred, but the child is still living within the home (i.e. not placed in state care). The child’s level of risk will be judged according to Levels 2 to 3 in line with the guidance contained in the document entitled ‘ *Thresholds for referral to Tusla Social Work services*’ ( Tusla, 2014). This document is based on the Hardiker model, which is widely used as a planning framework in child welfare and protection services in both the UK and the Republic of Ireland ( Hardiker *et al.*, 1991; see Supplementary Figure 1).
- Parents must be judged by child welfare professionals to be stable in terms of substance use or mental illness, i.e. parents must have a capacity to engage with the intervention.
- Parents/families must be willing and able to attend the services offered.
- Parents/families must agree to participate in the research. Children between 7–11 years must give assent to providing data; children below seven years are too young to provide data.

***Exclusion criteria***

- Families who display unstable substance use/mental illness.
- Parents who have had previous exposure to an evidence-based parent-training programme.
- Child is living in temporary or permanent out-of-home placement.

***Eligibility of programme providers***

In order to promote consistency of intervention delivery across sites and personnel, staff must:

- Have considerable experience in working within the child welfare and protection system in Ireland. i.e. formal recognised qualification e.g. NSWQ plus at least 5 years working directly within the child protection, early intervention and family support services in a senior role.
- Be trained and experienced in the delivery of the key components of the ChARM programme. For instance, all programme facilitators must be fully trained and accredited in delivering IYPP as well as having previous experience of delivering the programme with high risk families. Staff will also have direct experience of delivering individual home supports to vulnerable families. Staff training will be provided on PLSP prior to commencement of the programme as well as regular peer support coaching.

### Recruitment

We aim to recruit approximately 50 families over a period of 24 months (2015–2017) at the two participating centres. Potentially eligible families will be identified using existing waitlists within each site, as well as through liaison with a range of other statutory and community-based services in the area, who may also refer potential participants to the participating sites. Voluntary self-referrals will be accepted if the participating site deems that the family meets the inclusion criteria for the study. Many of the families involved in the study will most likely have an allocated social worker. Each site will meet with eligible families to discuss the intervention and the research evaluation. Families will be given a brief information sheet inviting them to receive further information about the study, and requesting that they provide their consent to forward their contact details to the research team. Participants will then be contacted by telephone to arrange for the research interviewer to visit them at home and to inform them about the study and obtain their written informed consent. Written informed consent will be obtained before any study-specific procedures, including collection of baseline data. Families will be thanked for their time and given a shopping voucher worth €20 at each data-collection visit. Collectively, the research team have considerable experience of working with vulnerable and difficult-to-engage populations and their expertise, in conjunction with the advice and support of the collaborators, will be important in managing the recruitment process.

### Procedure

**Figure 1.**
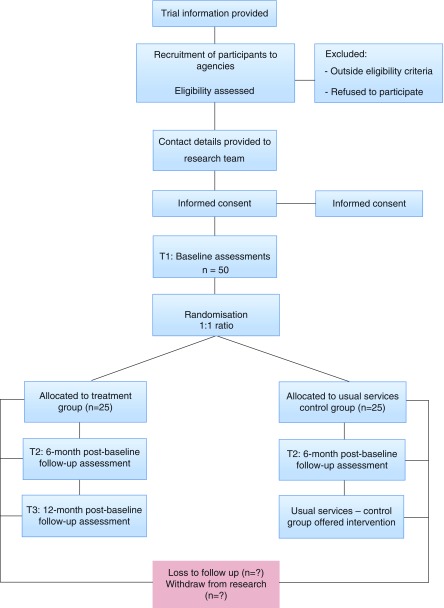
Study Flow Diagram.

***Study design.*** The ChARM study is a randomised controlled, parallel group, investigator-blinded, superiority trial (n = 50) comparing the ChARM intervention with usual services (1:1 allocation ratio), and a primary endpoint of incidences of child maltreatment and child wellbeing at six-month follow up. Data will be collected at three time points: T1 (pre-intervention), at six-month follow up (T2; one-month post intervention), and at 12-month follow up (T3). Assessment of the control group will continue to T2, after which they will receive the ChARM programme. Assessment of the intervention group alone will continue to T3. We will follow CONSORT guidelines for reporting parallel group randomised trials ( Moher *et al.*, 2010). Figure 1 shows the study flow diagram.

The embedded process evaluation - in line with the guidelines of the Medical Research Council (MRC) - aims to develop a logic model of the ChARM programme, elucidating key processes in programme development and implementation, impacts and outcomes ( Moore *et al.*, 2014; see Supplementary Figure 2). Specifically, it will aim to:

•  Identify key programme content and perceived mechanisms of change;

•  Assess enablers and barriers to programme development and implementation within the trial;

•  Evaluate fidelity of delivery and participant engagement; and

•  Investigate the feasibility of implementing the programme among services not involved in the trial

The embedded costs analyses will include a cost effectiveness analysis (CEA) and a cost-benefit analysis (CBA). The CEA will be based on a societal perspective (involving public sector costs, and costs incurred by participants in attending the programme) and will assess the costs of delivering the ChARM programme compared to usual services. If the intervention demonstrates effectiveness, the CBA will investigate the down-stream impact of the intervention on later costs, such as generating savings in relation to reduction in child welfare services, foster and residential placements, health and mental health service utilization, crime, education and unemployment.

### Randomisation and blinding

Participants will be randomly assigned by an independent statistician (in the Northern Ireland Clinical Trials Unit [NICTU]) to either the ChARM programme or to standard services with a 1:1 allocation using a computer-generated randomisation schedule stratified by site using permuted blocks of random sizes. The NICTU will use sequentially numbered, opaque, sealed envelopes to conceal the randomisation code until the participant has been recruited into the trial, which will take place following completion of baseline assessments. Block sizes will be concealed throughout the duration of the study. Throughout the study, randomisation will be conducted by the NICTU in order to keep the data management and the statistician blind against the study condition as long as the data bank is open. The randomisation list remains with the NICTU for the duration of the study. Thus, randomisation will be conducted without any influence of the principal investigator, data collectors or practitioners delivering the intervention.

Follow-up assessments at T1 and T2 will be performed by research staff blinded to study arm. At T3, we will only collect data from intervention families so blinding will not be relevant. At T2, participants will be requested not to disclose their group allocation to the researcher. If unblinding occurs, another assessor will be brought in to re-establish blindness. Any evidence of unmasking of blinding will be taken into account at the analysis stage. Due to the nature of the intervention, neither participants nor practitioners can be blinded to allocation.

### Contamination

To reduce the risk of contamination between the intervention and control participants within sites, staff who deliver the ChARM intervention will not be involved in delivering usual services to families in the control group. In addition, practitioners in both the intervention and control groups will be asked about the extent to which they shared with each other/learned of content from the ChARM programme and passed this information to families in the control group. If levels of contamination are found to be high in the control group, an extra confounder variable denoting contaminated controls will be added to the analysis and the effects of this contamination investigated.

### Intervention

**Figure 2.**
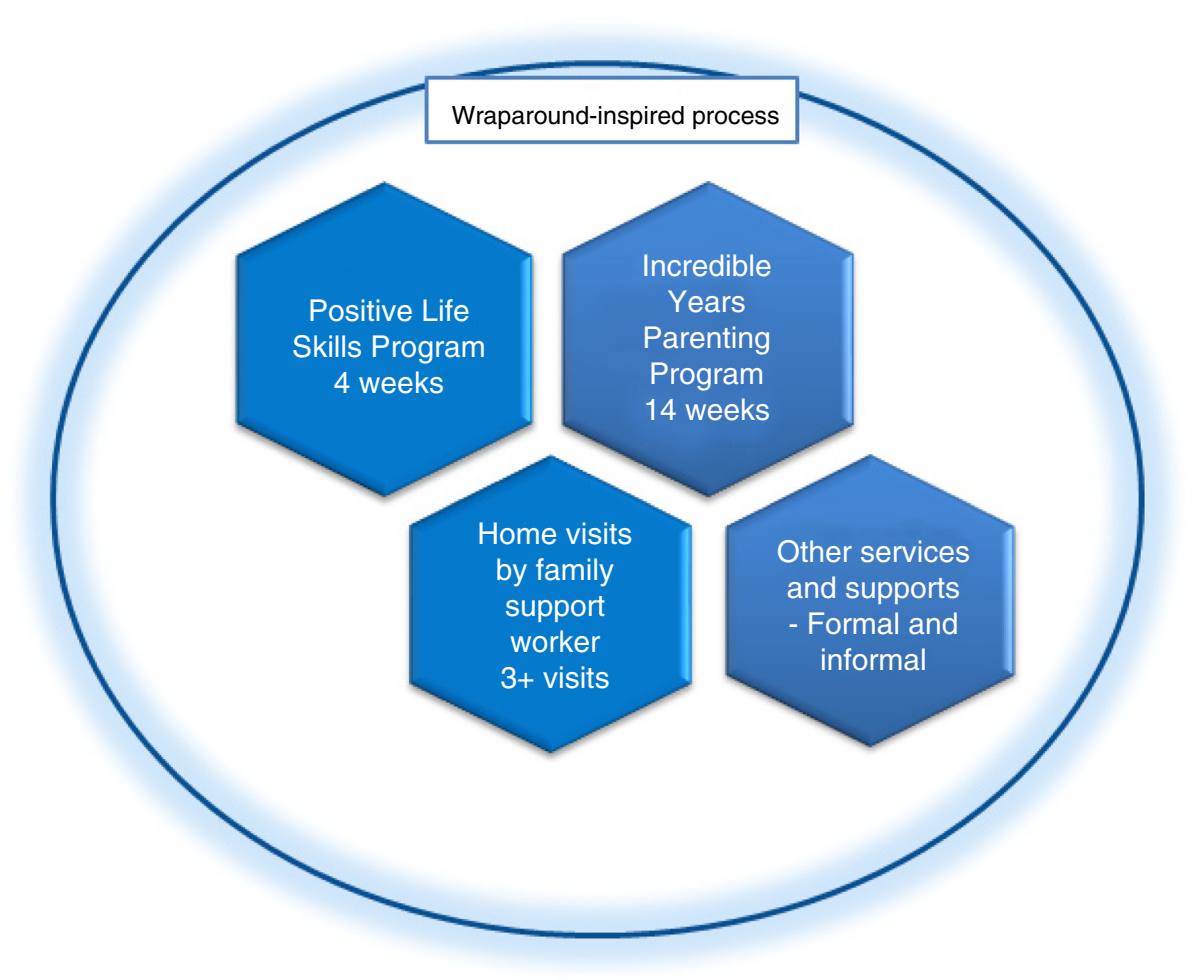
Core Components of ChARM Programme. ChARM involves an intensive package of supports for families inspired by a wraparound philosophy of care. It comprises core components of parent training, home visits, a positive life skills programme and additional supports as desired by families.

The ChARM programme involves the coordination of three ‘core’ components, as well as additional services and supports (formal and informal) that will be provided to families, as necessary (See Figure 2). The core components include: (1) the Positive Life Skills Programme (PLSP); (2) the Incredible Years Parenting Programme (IYPP); and (3) home visits. Both the PLSP and home visits may be used to initially engage families, although not all families will require home visiting as a means of engagement. Home visits will be conducted concurrently with the delivery of the PLSP and the IYPP. The programme will last 20 weeks. More details on the programme components are provided below.

***Coordination of supports.*** Each family will be already linked to a caseworker (social worker, family support worker) informed of the wraparound approach. The caseworker will discuss the suitability of the ChARM intervention with the family. Families must consent to engage with the three core components of the programme. Family strengths and needs will be examined and families will have an opportunity to identify other services and supports, besides the three core components, that may help them to achieve their goals. If any issues emerge during the family’s participation in the ChARM, additional services will be provided/recommended. The caseworker for intervention families in this study will also be a facilitator of the group programme s within the intervention.

***The Positive Life Skills Programme - PLSP.*** The PLSP is a manualised four-week, two-hour, parent-group programme, developed as a brief intervention to encourage vulnerable, hard-to-reach parents to engage with services. Many ‘at risk’ families suffer from mental health, addiction and other issues and consequently, parents may not possess the skills and self-esteem to engage constructively with needed services and supports. Sessions are delivered by two group facilitators who are trained in programme delivery. The four sessions help parents to: engage in a group setting with other parents and with service providers in a therapeutic space that allows sharing of personal issues; develop confidence, self-esteem and resilience in engaging with services; and build skills for daily living, including developing communication, stress and conflict management skills.

***The Incredible Years Parenting Programme – IYPP.*** The IYPP is a well-known evidence-based parenting programme that has demonstrated effectiveness in improving child emotional and behavioural problems, and parental mental health, within high-risk populations ( Furlong *et al.*, 2012). Recent studies of a clinically-informed adaption of the programme for families within the child welfare system have indicated preliminary evidence for improved parenting practices ( Hurlburt *et al.*, 2013; Letarte *et al.*, 2010). The IYPP consists of 14 weekly, 2-hour, parent-group training sessions, and topics include: learning to play with the child; social and emotional coaching methods; increasing positive behaviour through praise and incentives; problem-solving; and managing non-compliance and aggression through limit setting, ignoring, and other strategies. The sequence of topics for child welfare populations is similar to standard IYPP protocols, but has a greater focus on parent-child attachment, emotional and social coaching, parental attributions and self-talk, monitoring and self-care, along with increased dosage and home visits, if necessary ( Webster-Stratton & Reid, 2010). Sessions use dvds, role-play, modelling, group discussions, homework assignments and mid-week phone-call support to help parents rehearse and adopt positive parenting strategies. The IYPP addresses access issues and advocates provision of transportation, childcare and meals to parents. The programme also encourages parents to set up peer networks outside of group sessions in order to promote connections to the community and to increase the self-sufficiency of parents ( Webster-Stratton & Hancock, 1998). Within the ChARM programme, the IYPP will be delivered following the PLSP.

***Home visits.*** Home visits will be provided in parallel to the delivery of the PLSP and the IYPP, although in some cases, families will receive home visits before the PLSP in order to engage them to the ChARM programme. Family support workers will visit family homes and coach parents in positive parenting practices. Home-visiting sessions will reinforce the positive parenting principles taught in the IYPP using similar content, role-play and vignette strategies, as outlined in the IY home-visiting coaching model ( Lees *et al.*, 2014). They may also link families into other services, teach them how to complete housework or to seek social support when necessary, such as in transporting children to activities. The number of home visits per family will vary, as some families will require significantly more assistance than others. We will document the number of home visits received by families.

***Additional supports.*** Families at risk of CM present with a number of complex needs, including: substance abuse, mental health problems, health difficulties, educational deficits, unemployment, child disabilities, and so forth. The components outlined above may not be able to deal effectively with these issues. Consequently, caseworkers will collaborate with families in order to help them engage with relevant community-based agencies to address such issues. The additional supports may include, but are not restricted to, outreach activities, resilience and social skills training, housing and financial advice, referral to a substance abuse clinic, therapeutic services for family members, and so forth. Families will also be encouraged to utilize informal supports. The type and frequency of services and supports received by families will be documented as part of the costs and process evaluations conducted within the context of this study.

***Services as usual.*** Standard services will be provided by the child welfare and protective system in Ireland and may vary by site and family need. Families in the comparison condition will be assigned a caseworker who will arrange referrals to appropriate services as required, e.g. referral to substance abuse clinic or adult mental health centre. The type and amount of services received by families in the control condition will be documented by the research team. Families in the control group will be offered the ChARM programme at T2, i.e. at six-month follow up.

### Sample size

Due to major restructuring of services and staff within the Tusla Child and Family Agency in 2014–2016, our key collaborating site had to withdraw from the research. Thus, our sample size will be smaller (n = 50) than that advised by our sample size calculation that indicated that, factoring in 30 per cent attrition, we would need to recruit 150 families to detect a 0.8 effect size on our primary outcome measures. Given the reduced sample size, the results of this RCT should be interpreted with some caution.

### Measures

A range of psychometrically robust measures will be administered to all participants who agree to take part in the ChARM research programme. These measures have been selected because they are brief and easy-to-complete whilst also providing as comprehensive an assessment as possible of all participating families. Table 1 and Table 2 outline the measures used within the RCT, process evaluation and costs analyses. Further details on the outcome measures used are also available in the Supplementary Material ( Supplementary File 1).

**Table 1.** Measures within the RCT. A list of psychometric and observational measures will be administered as part of the impact evaluation to assess outcomes for families.

Measure	Participant	Objective
Impact evaluation		
Conflict Tactics Scale Parent- Child – Short Form	Parent	Parent-reported incidences of child maltreatment
Strengths and Difficulties Questionnaire adult version	Parent	Parent report of child behaviour and wellbeing: conduct, peer & emotional problems, hyperactivity
Strengths and Difficulties Questionnaire child version	Child 7–10 years	Child report of own behaviour and wellbeing: conduct, peer & emotional problems, hyperactivity
Brief Child Abuse Potential Inventory	Parent	Risk factors for child abuse, e.g. parental distress, rigidity, problems with child, self, family and others
Parenting Stress Index	Parent	Parenting stress and parent-child relationship
HOME SF 3–5/6–10 years	Parent and child	Observation of parent-child interaction in the home
Depression, Anxiety and Stress scale	Parent	Parental depression, anxiety and stress
CAGE	Parent	Screener for alcoholism of parent and partner
Drug Abuse Screening Test	Parent	Drug use of parent and partner
Record of incidence of child maltreatment	Collaborating site	Social work record of incidence of child maltreatment in previous six months
Record of out-of-home placement	Collaborating site	Social work record of incidence of out-of-home placement in previous six months
Profile Questionnaire	Parent	Demographic information on families

**Table 2.** Measures within the process evaluation and economic analyses. The process evaluation will utilize a range of qualitative and quantitative measures to assess programme fidelity and implementation, recruitment of sites and families, participant engagement and experiences, and the feasibility of implementing the programme within child and family services in Ireland not involved in the RCT. Several measures will also be applied in order to conduct a costs analyses of ChARM.

Measure	Participant	Objective
Process evaluation		
Session checklists	Practitioners	Fidelity of program content
Work Alliance Inventory	Practitioners & Parents	Practitioner-parent relationships
IY Agency Implementation Effectiveness Questionnaire	Practitioners & managers	Site and practitioner capacity to implement the program with integrity
In-depth semi-structured interviews	Practitioner & managers	Assess experiences of developing, coordinating and implementing program
Records of meetings	Research team	Assess experiences of recruiting sites, developing and implementing program
Attendance records	Practitioners	Records of parental attendance to program
PLSP feedback form	Parent	Parental feedback on Positive Life Skills Program
Home visits feedback form	Parent	Parental feedback on home visits
IY parent satisfaction questionnaire	Parent	Parental feedback on Incredible Years parenting program
Working Alliance Inventory	Parent	Parent-practitioner relationship
Semi-structured interview for parents (including attritors)	Parent	Assess experiences of participating in the program
Draw and Tell interview	Child 7–11 years	Experiences of child wellbeing and family
Cantril’s ladder	Child 7–11 years	Life satisfaction on 1–10 scale of ladder
My family and me	Child 7–11 years	Emotional closeness of family relationships
Semi structured interview/focus group	Child and Family services	Assess feasibility of implementing the ChARM program within current systems of care in Ireland
Economic analyses		
Costs diaries for program inputs	Practitioners & managers	Estimate the cost per family of delivering the program
Service Utilisation Questionnaire	Parent	Document health, educational and social services used by families in previous six months

***RCT***

The trial has two primary outcomes:

- Parent-reported incidences of child maltreatment, assessed with The Conflict Tactics Scales Parent-Child – Short Form Amended (CTSPC – SFA) ( Straus *et al.*, 1998). The CTSPC-SFA measures incidences of psychological aggression, neglect and non-violent discipline, and threats of corporal punishment. The parent will complete the CTSPC-SF for a chosen index child and sibling.
- Child behaviour and wellbeing, assessed using both the parent- and child-report versions of the Strengths and Difficulties Questionnaire (SDQ) ( Goodman, 1997). The SDQ assesses child conduct problems, hyperactivity, emotional symptoms, peer problems, and pro-social behaviour among 3–17 year olds. Parents will complete the SDQ for a chosen index child. The child-report version of the SDQ is appropriate for administration to children seven years and above; therefore, it is will be administered to a subsample of children within this study, i.e. children aged 7–10 years ( Di Riso *et al.*, 2010).

Secondary outcomes are:

- Risk factors for child abuse (Brief Child Abuse Potential Inventory [BCAPI]: parent report; Ondersma *et al.*, 2005);
- Parenting stress and parent-child interaction (Parenting Stress Index – Short Form [PSI-SF]: parent report; Abidin, 1995);
- Observation of parent-child relationship in the home environment (Home Observation for Measurement of the Environment Short Form [HOME-SF]; Caldwell & Bradley, 2001);
- Parental depression and anxiety (Depression, Anxiety and Stress Scale – Short Form [DASS-SF]: parent report; Lovibond & Lovibond, 1995);
- Parental alcohol and drug use (CAGE and the Drug Abuse Screening Test - 10 [DAST-10]: parent reports; Ewing, 1984; Skinner, 1982); and
- Child welfare reports of CM and out-of-home placements, assessed by records within the collaborating sites.

Demographic and background information on families and children will be collected by means of a Profile Questionnaire. Details on socioeconomic status (SES), and risk of CM, will be collated from questions on, for example, parental age, health, marital status, education and employment, living circumstances, child health, and so forth. Data for all outcomes will be collected at baseline, 6- and 12-month follow ups by a researcher who will meet with the participant in the family home, or, if preferred, in a local family/health care centre.

***Process evaluation.*** The process evaluation will utilize a range of qualitative and quantitative measures to assess programme fidelity and implementation, recruitment of sites and families, participant engagement and experiences, and the feasibility of implementing the programme within child and family services in Ireland not involved in the trial ( Table 2). Fidelity and implementation will be assessed with: weekly session checklists of all key components; practitioner capacity to engage parents (Work Alliance Inventory short form; Hatcher & Gillaspy, 2006); site and practitioner capacity to implement the programme with integrity (adapted version of the IY Agency Administration Implementation Effectiveness Questionnaire; Webster-Stratton, 2014); and in-depth semi-structured interviews with practitioners and managers following programme delivery. Records of meetings, training, certification and receipt of supervision will also be documented.

Parental engagement and experiences will be assessed using: attendance records; parental feedback on key intervention components (e.g. the Incredible Years Parent Satisfaction Questionnaire); the Work Alliance Inventory short form that measures a participant’s experience of the practitioner ( Hatcher & Gillaspy, 2006); and an in-depth semi-structured interview with a purposive sample of participating parents (n = 15; selected based on site and demographic characteristics, including those who dropped out from the intervention). Brief interviews will also be conducted with children aged 7–11 years at baseline and 6-month follow up in order to assess the impact of the programme on their perceptions of family relationships and their own wellbeing. The child measures include: the Draw and Tell technique ( Merriman & Guerin, 2007), Cantril’s My Life Ladder ( Cantril, 1965) and My Family and Me ( Hill *et al.*, 1996).

We will also conduct interviews/focus groups with a range of child and family services nationally (n = 30 organisations) in order to investigate the feasibility of implementing the ChARM programme within current systems of care in Ireland. This is important in light of the difficulties experienced in retaining collaborating sites as part of the RCT.

Interviews will be conducted in the participants’ home/place of work or a local health care centre. Participants can elect whether to participate in an individual interview or a focus group. Written informed consent will be requested. Interviews will be audio-recorded (with participants’ consent) and will last no more than one hour with parents and service providers, and no more than 30 minutes with children. The parent of the child will be approached to seek their consent for their child to participate in the study and we will also seek the child’s written and verbal assent. To reduce participant burden, interviews with parents and children will be conducted at a different time from the administration of the measures for the impact evaluation.

***Costs analyses.*** In order to estimate the costs per family of delivering the ChARM programme, comprehensive cost diaries will be completed by sites (practitioners and managers) during and following the implementation process. Costs will be collected on: costs of training and supervision, staff time and materials involved in preparation, recruitment of families, intervention delivery, managerial overheads, referrals, and so forth. Parents (n = 50) will also complete a Services Utilization Questionnaire (SUQ) at baseline and 6-month follow up in order to record all health, educational and social services used by the family in the previous six months. The SUQ is based on an adaptation of the Client Service Receipt Interview ( Beecham & Knapp, 1992).

### Data analysis

***RCT.*** Changes in continuous primary and secondary outcomes at baseline and at six-month follow-up will be compared for the intervention and control groups using ANCOVA, controlling for intervention status, site, baseline score and any other baseline differences identified. Mean difference effect sizes, 95% confidence intervals (CIs), and p values will be reported for continuous outcomes. Changes at 12-month follow up will be conducted using ANOVA. Changes between study arms in categorical variables (i.e. data records of incidences of CM and out-of-home placements) at baseline and six-month follow up will be analysed using the Chi Square test of independence, reporting relative risk, 95% CIs and p values. Descriptive statistical summaries (e.g. means, standard deviations, frequencies) will be presented for primary and secondary outcome measures at each time point. All data for primary and secondary outcomes will be analysed using an intention-to-treat analysis, using multiple imputation (MI) to compensate for missing data at different assessment points. Imputation assumptions for MI will be reported and justified, and imputed data analysed as part of a sensitivity analysis. Parallel per protocol analyses will also be conducted for outcomes. Attrition analyses will be conducted at each time point to assess for differences between those who dropped out from the programme me and those who stayed. This will be based on an examination of key baseline variables (e.g. intervention arm, participant SES and wellbeing, child gender) and qualitative data outlining reasons for attrition.

Multiple regression techniques will be used to explore moderators of intervention effects (if present). Moderators will include: severity of risk and CM at baseline (measured using below and above clinical cut-off scores on the BCAPI, CTSPC, as well as frequency of CM incidences within substantiated reports); age and SES of parents and children (measured using a composite risk factor score derived from demographic data on the Profile Questionnaire); gender of child; parental mental health and problem substance use (using above or below clinical scores on the DASS, CAGE and DAST). Statistical analyses will be conducted using SPSS and Stata. We are aware of the possibility of low statistical power given that our numbers are lower than desired. Hence these analyses will be more exploratory in nature.

***Process evaluation.*** Quantitative assessments of programme fidelity and participant engagement/satisfaction will be assessed using descriptive statistics and using correlational and regression techniques, where necessary. Interview data will be fully transcribed and coded using the qualitative analysis software package MaxQDA ( MaxQDA, 2016). Key themes and subthemes will be identified using framework analysis, a method suitable for applied policy research that has specific questions, a limited period, a pre-designed sample and a priori issues ( Ritchie & Lewis, 2003). Analysis of themes will be informed by the MRC framework, and will identify programme and implementation processes, contextual factors, mechanisms of impact, and intended outcomes ( Moore *et al.*, 2014). Framework analysis uses five steps to identify themes: familiarization; identifying a thematic framework; indexing; charting; and mapping and interpretation ( Ritchie & Lewis, 2003).

For the child measures, drawings will be analysed using Visual Content Analysis (VCA), which is a technique for systematically describing written, spoken or visual communication ( Bell, 2001). Analysis of the drawings will involve coding for common themes/categories, such as who is present in the picture (peers, family, friends, or pets); the setting (such as watching TV or playing outside); use of colour; and facial expressions (e.g. happy or sad). Data from the VCA will be supported by data from the audio-recordings used in each child interview in order to thematically analyse the child’s perception of their life and family relationships.

***Economic evaluation.*** A societal perspective (public sector perspective and individual costs incurred by participants in attending the intervention) will be taken in the economic analysis. The CEA will be calculated through a three-step process. Firstly, the costs diaries will estimate the cost per family of delivering the programme. Unit costs of health and social care services used by families (e.g. GP, nursing, hospital visits) will be obtained from official government documentation, official government pay scales, the Casemix/HIPE unit of the Health Service Executive and any other relevant sources and/or agencies. Thirdly, a CEA will calculate an incremental cost-effectiveness ratio (ICER) to give the cost of obtaining a one-unit decrease on the two primary outcome measures (CTSPC-SF and SDQ) when comparing the ChARM programme to usual services at six-month follow up.

The ICER will use a 1000 replication bootstrap to provide a 95% CI accompanied by appropriate sensitivity analyses. Such sensitivity analyses may include how the ICER may vary according to the severity of the presenting problem at baseline or, for example, excluding non-recurrent costs (e.g. training, materials). The ICER accommodates sampling (or stochastic) uncertainty and varying levels of willingness to pay for reductions in the primary outcomes of interest.

A CBA will also be conducted to investigate the down-stream impact of the intervention on later costs, such as generating savings in relation to reduction in child welfare services, foster and residential placements, health and mental health service utilization, crime, education and unemployment. To conduct the CBA, the results of the CEA will be combined with estimates of the effects of CM on key outcomes in adult life. The effects of CM on adult outcomes can be assessed using secondary data sources and a monetary value will be assigned to the associated gains/losses of programme me delivery. The CBA will calculate an ‘internal rate of return’ to assess the desirability of investment in the programme. The ‘internal rate of return’ refers to the discount rate at which the value of the stream of future benefits exactly equals the initial cost of the programme, yielding a net present value equal to zero.

## Discussion

The prevention of child maltreatment (CM) is an important public health priority and not least due to its negative impact on long-term personal, social, and economic outcomes. Although a range of interventions have been developed to prevent child abuse and neglect, even the most promising fail to engage families most at risk, or are targeted only at very young children (0–3 years). This study will evaluate the ChARM wraparound-inspired intervention, which incorporates evidence-based programmes and community-based supports in order to address the multiple and complex needs of vulnerable families whose children are aged 3–11 years. Furthermore, key process and implementation mechanisms of the programme will be investigated. The study is the first evaluation of a wraparound-inspired programme designed to prevent child abuse and neglect. Therefore, the findings will provide unique and valuable insights into the development and implementation of programmes designed to prevent child abuse and neglect.

However, some of the study limitations must be recognised. For example, the results, when they become available, should be treated with caution due to the small-scale nature of this exploratory RCT. In addition, while RCTs are the current standard for evidence-based practice, there have been recent debates on the utility or otherwise of RCTs ( Deaton & Cartwright, 2018). Notably, however, we will be using other methods alongside the RCT in our evaluation. Furthermore, ChARM does not offer all of the ingredients or flexibility to account for the chaotic lives of some families who continue to face major problems such as housing, relationship and/or addiction issues. It is likely that addressing the multiple needs of such high risk families will require more intensive supports over a longer period of time.

### Trial status

The study is in the process of collecting data.

### Compliance with ethical standards

**Ethical approval:** All procedures performed in studies involving human participants will be in accordance with the ethical standards of Maynooth University’s Social Research Ethics Committee (Reference number SRESC-2015-005, approved 16.02.2015) and with the 1964 Helsinki declaration and its later amendments or comparable ethical standards ( World Medical Association Declaration of Helsinki, 2013). The research will also be conducted in strict accordance with the ethical codes of conduct of the British Psychological Society and the Psychological Society of Ireland. Due attention will be paid to the core principles of beneficence, non-maleficence, autonomy and inclusivity, whilst the reporting of all data as well as the conduct of the one-to-one interviews, will be undertaken with particular care (e.g. using pseudonyms). The nature of the research is such that ethical considerations must be paramount at all times and will be monitored closely by the team throughout all stages of the study.

**Informed consent:** Informed consent will be obtained from all individual participants in the study. Children over seven years will be asked to give their verbal and written assent where parental written informed consent has first been obtained.

**Confidentiality and data protection:** All data will be anonymized and will not be identifiable. Data will be encrypted and uploaded to a secure, central site to which only members of the research team will have access.

**Study withdrawal:** All participants will be informed that they may withdraw from the study, and/or withdraw their data, at any point without affecting their access to services.

**Child welfare:** In the event that any child protection issues will emerge either directly or indirectly in the course of the research, these will be dealt with sensitively, promptly and in line with established guidelines for the protection of children (e.g. Children First: Natonal Guidelines 2017; Our Duty of Care, 2002) with referral, where appropriate, to a relevant HSE agency or in an emergency to the Gardai ( DCYA, 2011).

### Other ethical issues

The research team recognises that it has a duty of care to individuals with whom they may come into contact for research purposes. It is possible that issues of mental or physical wellbeing may arise for participants and/or their children. If necessary, parents or children will be referred to a contact within the recruiting agency, with whom we will be working closely throughout this research. Parents and/or children will also be ‘signposted’ to other services/supports, should the researcher have any concerns during the assessments. Parents may also indicate their preference for a project worker to be present. Access to parents will be facilitated by social work practitioners who are working with the research team.

## Data availability

No data is associated with this article.

## Supplementary material

**Supplementary Figure 1: The Hardiker model ( Hardiker *et al.*, 1991).** A planning framework widely used in child welfare and protection whereby a child’s level of risk is judged according to levels of risk 1–4. Among inclusion criteria for participants in this study is whereby a child’s level of risk is between levels 2–3.

**Supplementary Figure 2: Medical Research Council (MRC) framework for process evaluations ( Moore *et al.*, 2014).** This figure outlines the key functions of process evaluation and relations among them. MRC guidance provides a framework for conduction and reporting this process evaluation study.

**Supplementary File 1.**

Details on the outcome measures used
