# Supplementary material for: A community-based parent-support programme to prevent child maltreatment: Protocol for a randomised controlled trial
Source: HRB Open Res. 2018 Sep 21;1:13. Originally published 2018 Apr 5. [Version 2] doi: 10.12688/hrbopenres.12812.2 (PMC6973527; doi:10.12688/hrbopenres.12812.2)
Supplement: Supplementary file 1 [file hrbopenres-1-13945-s0000.tgz › 75497569-6d41-48b8-9ad6-84bfb2587324.docx]

**Supplementary Figure 1: The Hardiker model (Hardiker *et al* 1991).** A planning framework widely used in child welfare and protection whereby a child’s level of risk is judged according to levels of risk 1-4. Among inclusion criteria for participants in this study is whereby a child’s level of risk is between levels 2-3.

**The Hardiker Model**

The Model outlines four levels of intervention as follows:

**Level 1:**Level 1 refers to mainstream services that are available to all children — health care, education, parent and toddler groups, leisure and a range of other services provided in communities.

**Level 2:**Level 2 involves delivering more targeted services to children/families who have additional needs. Services at Level 2 are characterised by referral, and full parental consent and negotiation. Examples include: parenting support and additional educational services for families who are deemed vulnerable through an assessment of needs.

**Level 3:**Level 3 provides support to families or individual children/young people where there are chronic or serious welfare and wellbeing issues. Support is often provided through a complex mix of  services which usually need to work together well in order to provide optimal support. State intervention may be necessary at this level. Examples include children being placed on the Child Protection Register or who have come before the Courts.

**Level 4:**Level 4 represents support for families and individual children/young people where the family has broken down temporarily or permanently, and where the child may be looked after by social services. It can also include young people in youth custody or prison or as an in‐patient due to disability or mental health problems.
