# Supplementary figures and images for: A community-based parent-support programme to prevent child maltreatment: Protocol for a randomised controlled trial
Source: HRB Open Res. 2018 Sep 21;1:13. Originally published 2018 Apr 5. [Version 2] doi: 10.12688/hrbopenres.12812.2 (PMC6973527; doi:10.12688/hrbopenres.12812.2)

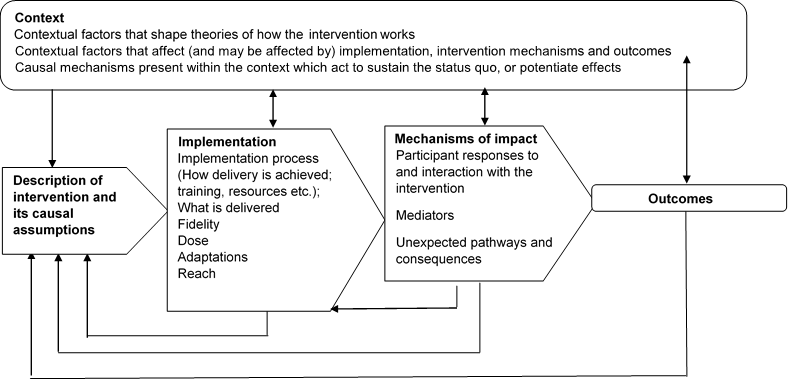

Supplement: Supplementary file 2 [file hrbopenres-1-13945-s0001.tgz › 85d0a7a2-b9ec-4572-b322-36edac47493b.png]
