# Supplementary material for: A community-based parent-support programme to prevent child maltreatment: Protocol for a randomised controlled trial
Source: HRB Open Res. 2018 Sep 21;1:13. Originally published 2018 Apr 5. [Version 2] doi: 10.12688/hrbopenres.12812.2 (PMC6973527; doi:10.12688/hrbopenres.12812.2)
Supplement: Supplementary file 3 [file hrbopenres-1-13945-s0002.tgz › 07698adc-ce1d-475a-9d00-6ac962973686_supp_file_1.pdf]

Supplementary File 1: Details on the outcome measures used

Primary Outcome Measures

- Parent-reported incidences of child maltreatment will be assessed using an adapted version of the *Conflict Tactics Scales Parent-Child – Short Form Amended* (CTSPC – SFA) (Straus et al. 1998). The original scale is is a self-report, 35-item instrument intended to measure psychological and physical maltreatment and neglect of children by parents, as well as enquiring about the parent's own experiences as a child. It measures the extent to which a parent has carried out specific acts of physical and psychological aggression, regardless of whether the child was injured. Internal reliability ranges from .70 to .80. NB. Due to legislation that was put in place in Ireland at the beginning of this study, (which banned physical punishment of children by parents), this measure was adapted to remove questions pertaining to the use physical assault or corporal punishment. Thus, an amended 12-item scale will be used which measures incidences of psychological aggression, neglect and non-violent discipline, and threats of corporal punishment. The measure takes 5-8 minutes to complete.

- Child behaviour and wellbeing will be assessed using both the parent- and child-report versions of the widely used Strengths and Difficulties Questionnaire (SDQ) (Goodman 1997). The SDQ is a 25-item, widely used and psychometrically sound behavioural screening measure for 3-16 year-olds. It comprises five subscales designed to assess child conduct problems, hyperactivity, emotional symptoms, peer problems, and pro-social behaviour. The respondent is asked to rate, on a three-point Likert scale, the extent to which a given item (or behaviour) (e.g. considerate of other people's feelings) is 'not true' (0) to 'certainly true' (2). The scores on each subscale (except the prosocial scale) may be summed to generate a 'total difficulties' score. Parents will complete the SDQ for a chosen index child. SDQ symptom scores may be used as continuous variables or classified as within 'normal' range, 'borderline' or 'abnormal'. The questionnaire will be completed by parents of the child and takes 5-8 minutes to complete.
-
    - A sub-sample of children aged 7-11 will complete the child-report version of the SDQ ($\alpha = 0.7$) (Di Riso et al. 2010). For this study, the child SDQ contains 25 statements which have been re-designed for purposes of this study in a more 'child friendly' format to make it more accessible for children from 8 years upwards. The researchers are cognisant of the fact that, due to differences in behavioural, cognitive, social and emotional maturation, some children may require additional help when completing the measure. In this instance, the researcher will guide the child through the questionnaire and assist where possible, should any difficulties in comprehension arise (e.g. by calling out the statements and offering interpretation of the statements where necessary). To further facilitate interpretation, the statements listed in the questionnaire contain both the SDQ trait labels (e.g. 'I am restless') as well as behavioural descriptions (e.g. 'I cannot stay still for long'). This will allow even young children to complete the SDQ. Should the child have any literacy difficulties, the statements will be read aloud to them and the child will

indicate their response. Researchers will be guided by the child's knowledge and understanding of the statements outlined in the SDQ. Where significant difficulties in comprehension arise, or where child discomfort is evident, the child will not be asked to complete the SDQ. The self-report version will take approx.10 minutes to complete. The SDQ will be included as part of the child interview (process evaluation).

Secondary outcome measures.

- Risk factors for child abuse will be measured by the *Brief Child Abuse Potential Inventory* [BCAPI]: parent report (Ondersma et al. 2005) (α = 0.88). This measure is a 34-item self-report questionnaire developed to identify individuals at risk for child abuse. Risk factors for abuse in the BCAPI include parental distress, rigidity, unhappiness, problems with child and self, problems with family and problems from others. The scale also contains two subscales designed to detect socially desirable or random responding. BCAPI has a 9-item validity scale and a 25-item abuse risk scale. The internal reliability of the full CAPI abuse scale is high, with KR-20 correlation coefficients ranging from .92 to .96 and good test-retest stability of .91 and .83 for 1-day and 1-month intervals, respectively. BCAPI is highly correlated with the full CAPI, and has a good inter-item consistency of .88. The measure takes 5-8 minutes to complete.

- *The Parenting Stress Index – Short Form* (PSI-SF; Abidin 1995) (α = 0.86) will be used to measure parent stress and functioning. The scale comprises 36 items that measure the distress experienced by parents in their parenting role as well as dysfunctional parent-child interaction. It is a commonly used measure designed to provide a reliable and valid measure of the nature and extent of stress experienced by parents in their parenting role (of a child aged up to 12 years). It comprises four sub-scales - Parental Distress (PD), Parent-Child Dysfunctional Interaction (P-CDI), Difficult Child (DC) and Defensive Responding (DR). The PD subscale measures distress experienced by a parent in his/her parental role as a function of personal factors directly related to parenting (e.g. 'I feel trapped by my responsibilities as a parent'). The P-CDI subscale focuses on the attachment quality of parent-child interaction (e.g. 'My child rarely does things for me that make me feel good') whilst the DC subscale assesses the presence of basic behavioural characteristics that makes a child easy or difficult to manage (e.g. temperamental characteristics). Respondents are asked to respond on a five-item Likert scale, ranging from 5 ('strongly agree') to 1 ('strongly disagree'). The DR subscale is also included in order to assess social desirability bias. The measure can be completed in approximately 5-8 minutes. Scores for each sub-scale may be summed and a total 'stress' score then computed by summing the subscale scores. The PSI-SF has been shown to have good internal reliability (from .80).
- *The Depression, Anxiety and Stress Scale – Short Form (DASS-SF*; Lovibond and Lovibond 1995), a 21-item scale, will also be used to measure parental mental health functioning and wellbeing. The scale comprises three subscales, namely, depression (α = 0.9), anxiety (α = 0.82) and stress (α = 0.87).
- Parental alcohol and drug use will be measured using the *CAGE* (Ewing 1984) and the *Drug Abuse Screening Test - 10* (DAST-10; Skinner 1982) respectively (α =0.78).

The CAGE is a brief, four-item scale which assesses problematic drinking (α = 0.72). This measure will also be adapted to collect information from the primary respondent on problematic alcohol use by partners (α = 0.92). The DAST-10 (α = 0.78) is a brief, 10-item screening tool which assesses drug use, not including alcohol or tobacco use, in the past 12 months. Like the CAGE measure, this scale will be adapted to collect data on partner drug abuse in the previous 12-months.

Observational Measure

The *Home Observation for Measurement of the Environment Short Form [*HOME-SF] (Caldwell and Bradley 2001) will be used to assess parent and child interaction and relationships. Two versions of the HOME-SF will be used - the 3– 5-year-old version (α= 0.6), comprising 24 questions, and the 6-9-year-old version (α= 0.71), comprising 29 questions, both of which will be employed to provide an observational index of cognitive stimulation and emotional support in the home. A combined total score may be generated by dividing the total HOME-SF score for each age group by the number of items applicable to that group, generating a score representing the proportion of items endorsed per assessment. The same procedure will be undertaken to generate two subscale scores - cognitive stimulation and emotional support or both age groups combined.

Data records from agencies

Participating families may avail of several services during the course of the research. In order to investigate which services are associated with trial outcomes, and in order to calculate accompanying costs, permission has been obtained from collaborating agencies to collect data on the service utilisation of participants from the date of entry into the study. Data will not be collected on participant service utilisation prior to study commencement. Data will only be collected from collaborating agencies where parental consent has first been obtained.

At the six-month follow-up, we will ask agencies to complete a Service Utilisation Form for both the intervention and control parents in order to indicate the ‘in-house’ and other services used/accessed by parents since the date of entry into the study.
