# Supplementary material for: A community-based parent-support programme to prevent child maltreatment: Protocol for a randomised controlled trial
Source: HRB Open Res. 2018 Sep 21;1:13. Originally published 2018 Apr 5. [Version 2] doi: 10.12688/hrbopenres.12812.2 (PMC6973527; doi:10.12688/hrbopenres.12812.2)
Supplement: Peer review history

# Peer review: Reviewer response for version 2

**DOI:** 10.21956/hrbopenres.13945.r26833

**Published:** 2019-10-22

**Authors:** Melissa O'Donnell

**Affiliations:** 1 Developmental Pathways and Social Policy, Telethon Kids Institute, Nedlands, Australia; 2 University of Western Australia, Perth, Australia

This protocol is for a randomised controlled trial for a community-based parent support program to prevent child maltreatment. 

 The objective of this study is clear that this protocol has been developed to evaluate the effectiveness, cost effectiveness and process mechanisms of the 'Children at Risk Model' for vulnerable families who are at risk of maltreatment. 

 The authors appear to have addressed the main issues of previous reviewers. The small sample size will potentially effect the power of the study to detect statistical differences in the effectiveness of the program but this is acknowledged by the authors who have clarified the exploratory nature of this study.

 There are a few points of clarification I wish to raise:

1. ChARM programme will be compared to standard care. Does standard care involve the Meitheal model? It wasn't entirely clear as the authors state that it has not been sufficiently embedded and whether this may vary by the two areas involved in the study. 
2. Under Methods, Participants it states that the programme will be delivered within a social work department and a family resource centre - can the authors stipulate what services these are (e.g. government, non-government/not for profit) and if a social work department what kind of service is this based in health, mental health, child welfare?
3. The authors explain that the focus is on families with children aged 3-11 years due to the lack of evidence for the effectiveness of programmes aimed at families of children 3-11 years. The inclusion criteria states that participants are parents of children aged 3-11 years, however the exclusion criteria does not exclude parents who have a parent 0-3 years. Was this intentional? If children aged 0-3 years are included an additional assessment may be worth including, such as the BITSEA. This was included in the randomised control of the Parents Under Pressure Programme which was recently published - Drug and Alcohol Dependence, 2019, Jane Barlow. 

Overall the protocol is clear and there is sufficient detail. A strong evidence base using RCT's for interventions with families is essential to guide practice in child maltreatment prevention.

I confirm that I have read this submission and believe that I have an appropriate level of expertise to confirm that it is of an acceptable scientific standard.

# Peer review: Reviewer response for version 2

**DOI:** 10.21956/hrbopenres.13945.r26382

**Published:** 2019-03-19

**Authors:** Nuala Connolly

**Affiliations:** 1 Barnardos Ireland, Dublin, Ireland; 2 EU-CoE Youth Partnership Pool of European Youth Researchers, Dublin, Ireland

The authors have addressed fairly all matters arising in peer review.

 The reviewer accepts the authors' comments. It is also noted that the paper follows journal guidelines.

 The reviewer has no further comments.

I confirm that I have read this submission and believe that I have an appropriate level of expertise to confirm that it is of an acceptable scientific standard.

# Peer review: Reviewer response for version 1

**DOI:** 10.21956/hrbopenres.13872.r26304

**Published:** 2018-07-23

**Authors:** Nuala Connolly

**Affiliations:** 1 Barnardos Ireland, Dublin, Ireland; 2 EU-CoE Youth Partnership Pool of European Youth Researchers, Dublin, Ireland

**Abstract:**

 The abstract introduces the ChARM model, study type and sample size. The abstract also describes the outcomes and provides a rationale for the study. The study is multifaceted and ambitious. The stage of the study is not entirely clear; it is described as “being conducted”, while assessments “will take place”. While this could be clarified, overall the abstract is clear and well-written.

**Introduction:**

 The introduction is detailed and covers considerable ground on CM and associated risk and protective factors.

 The wraparound approach, and rationale for an approach inspired by wraparound principles that incorporates evidence based programmes is well-presented and concise.

 Evidence on home-based supports and parent training is subsequently introduced.

 The case presented focuses on why parenting programmes alone/ home-visiting programmes alone are insufficient in improving risk factors and reducing incidences of CM.

 The authors have not acknowledged that parenting programmes can be home-based. For example, the evidence provided on parenting programme attrition speaks to centre-based group parenting programmes. Likewise, the next paragraph begins with the assertion that “home-based interventions have more capacity than parenting programmes”.

 Meitheal is an important inclusion, but should be described as a national practice model, rather than a national policy initiative.

 Page 5, Para 3 & 4 have some typographical errors, with the word ‘me’ inadvertently inserted a few times.

 The objectives are ambitious – particularly given the statistical power of the sample size. The hypotheses will be difficult to prove, particularly where they depend upon a subsample (secondary hypotheses). This is acknowledged by the authors.

**Method:**

 The methodology provides a detailed description of the RCT. Inclusion and exclusion criteria are covered, in addition to eligibility of programme providers.

 The procedure is robust and described in detail, including randomisation, blinding and contamination. It is clear the authors have paid particular attention to this aspect of the methodology.

 The recruitment timeline is a little unclear stating that the aim is to recruit 50 families between 2015 and 2017. What stage is the study currently at?

 While the main limitation (sample size) is addressed under said heading, it would be helpful to have a limitations section.

**Ethics:**

 The number of assessment scales used is considerable, particularly in the context of vulnerable parents. This could be better addressed in the paper. While ethical compliance is described, aspects relating to the potential risk to parents are not addressed. Will the parents have the option of having a project worker present during the administration of the measures/ interviews? Who will gate-keep access to the parents? What support will be provided during and following data collection? Additional discussion would benefit the paper.

**Overall:** 

 Overall, this is an interesting, timely and informative article on a study that aims to evidence a particular type of wraparound approach. With some edits, it will make a valuable and important addition to the literature.

I confirm that I have read this submission and believe that I have an appropriate level of expertise to confirm that it is of an acceptable scientific standard.

# Response

**Published:** 2018-09-11

**Authors:** Ann Stokes

**Affiliations:** National University of Ireland, Maynooth, Ireland

The abstract introduces the ChARM model, study type and sample size. The abstract also describes the outcomes and provides a rationale for the study. The study is multifaceted and ambitious. The stage of the study is not entirely clear; it is described as “being conducted”, while assessments “will take place”. While this could be clarified, overall the abstract is clear and well-written.

**The description of this study is in the future tense because this is a protocol and indeed, at the time of writing, the study is still ongoing. Thus, the aim of this article is to outline the study methods and activities involved therein. We have now added a sentence in the abstract to make this more clear (lines 5-6, p.1).**

Introduction:

The introduction is detailed and covers considerable ground on CM and associated risk and protective factors.

The wraparound approach, and rationale for an approach inspired by wraparound principles that incorporates evidence based programmes is well-presented and concise.

Evidence on home-based supports and parent training is subsequently introduced.

The case presented focuses on why parenting programmes alone/ home-visiting programmes alone are insufficient in improving risk factors and reducing incidences of CM.

The authors have not acknowledged that parenting programmes can be home-based. For example, the evidence provided on parenting programme attrition speaks to centre-based group parenting programmes. Likewise, the next paragraph begins with the assertion that “home-based interventions have more capacity than parenting programmes”.

**We understand that some parent programmes are delivered in the home.  Home-based interventions in the context of this study, refer to supports provided in the home via individual home visits designed to supplement the group-based parenting intervention that forms part of the ChARM model. We have now clarified this point and amended the original sentence on p.4, para 1.**

Meitheal is an important inclusion, but should be described as a national practice model, rather than a national policy initiative.

**This has been revised as suggested on p.5, para 3.**

****

Page 5, Para 3 & 4 have some typographical errors, with the word ‘me’ inadvertently inserted a few times.

****

**These typos have been corrected as highlighted (removal of ‘me’) (p5., para 4; p.6, paras 3, 4,).**

The objectives are ambitious – particularly given the statistical power of the sample size. The hypotheses will be difficult to prove, particularly where they depend upon a subsample (secondary hypotheses). This is acknowledged by the authors.

Method:

The methodology provides a detailed description of the RCT. Inclusion and exclusion criteria are covered, in addition to eligibility of programme providers.

The procedure is robust and described in detail, including randomisation, blinding and contamination. It is clear the authors have paid particular attention to this aspect of the methodology.

The recruitment timeline is a little unclear stating that the aim is to recruit 50 families between 2015 and 2017. What stage is the study currently at?

**As indicated earlier, the description of the study is written in the future tense as this is a protocol; therefore, we do not feel it is appropriate to comment on the current stage of the project. However, we have amended the ‘trial status’ section at the end of the paper (indicating that at this stage, data collection has just been completed).**

While the main limitation (sample size) is addressed under said heading, it would be helpful to have a limitations section.

**The research team have followed the SPIRIT reporting guidelines in preparing this article, as recommended by HRB Open Access and in so doing, the paper does not stipulate a specific limitations section.  However, we have now included some brief information on limitations of the study on p.15, para 2.**

Ethics:

The number of assessment scales used is considerable, particularly in the context of vulnerable parents. This could be better addressed in the paper. While ethical compliance is described, aspects relating to the potential risk to parents are not addressed. Will the parents have the option of having a project worker present during the administration of the measures/ interviews? Who will gate-keep access to the parents? What support will be provided during and following data collection? Additional discussion would benefit the paper.

**The research team have followed the guidelines of HRB Open Access in preparing this article and in so doing, the paper follows the SPIRIT reporting guidelines in terms of ethical (amongst other) requirements.  However, we have now expanded the section entitled ‘ethical standards’ on pp.15-16 to try to address these important points. We have also added another section entitled ‘other ethical issues’ on p.16.**

# Peer review: Reviewer response for version 1

**DOI:** 10.21956/hrbopenres.13872.r26184

**Published:** 2018-05-22

**Authors:** Marie-Josée Letarte, Isabelle-Ann Leclair Mallette, Caroline Temcheff

**Affiliations:** 1 Department of Psychoeducation, University of Sherbrooke, Sherbrooke, QC, Canada; 2 School of Psychology, McGill University, Montreal, QC, Canada

This study presents a protocol for a program evaluation using a RCT. The program studied here aims to prevent child maltreatment through the coordination of evidence-based programs. This paper is interesting for several reasons:

1. The social pertinence of the program is obvious. The authors demonstrate how important it is to prevent child maltreatment before it occurs. We know that intervention is less effective when offered once maltreatment is present in the family. The authors also demonstrate that empirically-supported programs are useful, but none can prevent child maltreatment on its own since vulnerable families are so hard to reach.
2. A good description of the RCT design is provided. Everything seems to be planned for a proper RCT protocol, with several controls regarding the randomization and blinding. The description is clear and precise.
3. The number of aspects considered in the evaluation is vast. Indeed, the authors are planning to evaluate primary and secondary outcomes, the therapeutic processes, and the cost-benefit ratio. The acceptability and feasibility of the program outside the involved sites is also noteworthy. Though ambitious, these considerations are valuable since it is a new and pertinent initiative and the authors seem to be confident that they will be able to disseminate the program throughout Ireland once it is evaluated, through another national initiative.
4. It is to the credit of those involved in the Irish CPS to look for real solutions to child abuse and neglect and to rely on empirically-supported programs in order to do so

We believe that the project merits indexing despite some limitations. However, a few aspects could be improved. These suggestions are detailed below.

**Abstract**

 The abstract presents the different sections of the article. To avoid any confusion, authors should state in the first or second sentence that this paper presents the protocol for a study currently being carried out or will be carried out in the near future (i.e., results not available yet). It is also slightly unclear whether the study has begun at this stage or not. For example, in the abstract it is stated that the study “is being conducted” whereas in the methods it is stated “ The ChARM programme will be delivered…”

 Again, to avoid confusion, the last sentence should be rephrased at the future tense: "the findings will offer ..."

**Introduction**

 The introduction is a plea for the prevention of child abuse and neglect. The goal of the program is relevant, as this problem is of major concern for most societies.

 The authors make a good demonstration that intervention is less effective when offered once maltreatment is present in the family and that prevention programs should be developed. However, it is not that clear from the methods that they propose a prevention program since targeted families are involved in child protection for child maltreatment (some only at-risk for maltreatment, but some will have documented maltreatment already). The authors also demonstrate that empirically supported programs are useful, but none can prevent child maltreatment on its own since vulnerable families are so hard to reach. Another argument for such a program could be that child maltreatment is explained by multi-systemic factors.

 Even though we are not questioning the fact that child maltreatment has important consequences on the victims’ development, the study cited to support this link is about child maltreatment prevention in general (Sethi et al., 2013). Perhaps studies examining consequences of child maltreatment (primary or meta-analysis) would be more appropriate to support this link.

 The authors present the incidence of maltreatment and more specifically of its different forms. In order to do so, they report rates found in meta-analyses of self-reported incidences. Afterward, they provide data for substantiated abuse. It seems to be a good idea to report both since they do not provide exactly the same information. However, when it comes to presenting the rates of the different forms of maltreatment, a little nuancing would be appropriate. In fact, even if it is true that, according to the self-reported rates, neglect is less frequent than physical abuse, this seems to be an under-representation that may be related to the type of measure used.

 Again, in relation to incidence, it is not clear why the authors introduce rates from different countries. When it is written that “prevalence rates of CM are even higher in low and middle-income countries…”, it is unclear why this is relevant to the current study. Does the critique about the unreliable detection and surveillance in most countries apply to Ireland? The pertinence of this discussion is questionable and it is unclear how it reflects the Irish situation.

 The authors mention some protective factors of CM: knowledge of parenting, nurturing parenting skills, parental resilience, social network, etc. These are not protective factors, as protective factors must have made the demonstration they can interrupt or interact with risk factors. These may be viewed as favorable factors or positive factors that MAY prevent CM and promote child well-being.

 The most important problem with the introduction is that it does not introduce ChARM, but focuses on the WA model of care. We understand that this model is central as it demonstrates the value of an approach that consist of coordinating services around the family. It would be good to discuss strengths and limitations of this model and the need to incorporate empirically-supported programs in WA care. The authors also introduce Meitheal, another program, also inspired by WA. However, there are very few allusions to ChARM, which leads to some confusion. It seems important to discuss the WA model, however I think the authors need to place the focus more on ChARM and to demonstrate the value of this program. Perhaps the progress of the WA implementation in Ireland and Meitheal could be discussed in the discussion ?

**Objectives**

 Though the objectives are totally relevant, we do have a concern about a specific hypothesis: (1) the ChARM program will reduce parent-reported incidences of child maltreatment. This comment will be detailed in the method, but the administrative data is considered as a secondary outcome, which is not consistent with the introduction.

**Methods**

 Since we are now in 2018, and the recruitment was planned for 2015-2017, would it be possible to have an update and the authors to state the exact number of participants they recruited? The authors could then perform post hoc computations of statistical power.

 There is slight inconsistency in the targeted age of the children. Sometimes it is written 3-10 and sometimes it is 3-11. Please verify.

 In this section, it is said that the ChARM program prevents child maltreatment “within intact families”. We were not sure if the term “intact family” refers to families in which both biological parents are living together or family in which the child is not in out-of-home placement.

 The objectives of the study are relevant, clear and precise. However, the first hypothesis is not clear. Why should the parent-reported incidences of child maltreatment decrease? Is it that parents will report having less maltreating behavior after the program?

 The introduction convinced us that the program to be evaluated is a prevention program of maltreatment, implemented in the community with families that are at risk to commit child maltreatment. We were surprised to see that participants were actually followed by child welfare, including some that had already committed child maltreatment. This apparent inconsistency may be due to differences between child protective services in Ireland compared to our system in Quebec (if it is the case, a brief description of CPS in Ireland could help). Or maybe ChARM is not a prevention program but an intervention one.

 Can you explain on what basis the child welfare professionals refer a parent at risk of abuse or neglect?

 The first and third exclusion criteria seem to be redundant with inclusion criteria.

 The eligibility criteria for program providers to be part of the study is not precise enough. What do you mean by “considerable experience”? In what capacity are they working within CPS? How much experience is required in order to deliver the components? It could be interesting to quantify this experience in months/years.

 ***The most important problem with this protocol is the number of participants to the study. 50 (25+25) over two years, seem to be very few, especially after the authors mention that this is a prevalent problem. If probing practices are implemented in order to reach and engage families, how do you explain such a small n? An analysis of the statistical power is essential. Indeed, we have to know if the number of participants available in your study and the number of outcomes to consider make it possible to reach your goals. It would be disappointing for everyone that you come to the conclusion that you can not state on the effectiveness of ChARM since your negative results are due to insufficient statistical power.

 This will probably not be enough to test moderations. You state that 150 families would be necessary in order to detect a 0,8 effect size, which is quite big for this population and this context. Are you willing to accept that with 50, you wouldn’t detect a 0.8 effect size? I read that the authors are aware of this problem. But we think they really need to find solutions.

 Since the protocol is so complete, we are interested to know more about the way contamination will be measured and controlled statistically if needed.

 Since the age of the children show a wide range, what program is used from IY? The preschool or the school age basic program?

 We would like to know more about the name and number of "subscales" for each measure used. Could you also give some information on the psychometric value of the selected tools? I also notice that these are several measures for very few participants. What correction are you planning to use to compensate, knowing that Bonferroni is relatively conservative... In addition, I invite the researchers to question the potential impact of the number of measures on the recruitment of participants. Indeed, the number of questionnaires provided in the protocol could have an impact on the possibility of generalizing the results to all the very vulnerable parents who would have refused to participate in the research.

 The WAI is used to measure the capacity to engage parents. This is known to be a measure of working alliance. There is at least two versions of this questionnaire: parent and therapist. Why not use the parent version? Furthermore, when is it going to be completed? Some researchers have shown that the validity of this measure and its capacity to detect relevant working alliance is at its best around the third meeting. We invite the authors to have a discussion about this question of timing.

 We wonder why you presented maltreatment report by parents as a primary outcome, and CPS report of maltreatment as a secondary outcome. We believe that because the parents are followed in CPS, which is a pretty coercive context, they could be tempted to give answers to their advantage which can cause a social desirability bias to this outcome. That is why we also suggest the use the official CPS report of maltreatment in combination to the parent-reported maltreatment as primary outcomes.

 It is a very interesting idea to have interviews with those who abandon the program. I wonder how they will be recruited. If they do not agree to go through the program, it is not very likely that they will accept the go further with the research process. In addition, those who could accept would have specific characteristics to be considered.

 We are also very satisfied that the fidelity of implementation of the program that is described in this study. However, we question the validity of using it as a moderator, because to be eligible as a moderator, a variable should respect criteria of temporal precedence and independence with the program (Kraemer, Kiernan, Essex & Kupfer, 2008).

 Thus, many good ideas, but perhaps not the statistical power to accomplish all this!

**Discussion**

 Very short. More information regarding the anticipated limitations of the study would be helpful.

**Ethics Standards**

 We appreciated the section about how the authors are planning on complying with ethical standards. This section, like all others, is precise and detailed. It shows the authors’ concern to follow best practices in program evaluation with vulnerable clientele.

**General**

 There are some language errors or typos. In the second paragraph, it is written that “In Ireland, over 40,000 referrals of child welfare and abuse cases.” I suppose authors mean abuse and neglect cases?...

 On p.5, paragraph starting with “This study involves the evaluation of…” and in the next one starting with “The objectives of the study…”, there are many typo problems. Many little “me” are inserted, and I cannot explain why. See the whole text for other problems of this kind.

We confirm that we have read this submission and believe that we have an appropriate level of expertise to confirm that it is of an acceptable scientific standard, however we have significant reservations, as outlined above.

# Response

**Published:** 2018-09-11

**Authors:** Ann Stokes

**Affiliations:** National University of Ireland, Maynooth, Ireland

**Abstract**

The abstract presents the different sections of the article. To avoid any confusion, authors should state in the first or second sentence that this paper presents the protocol for a study currently being carried out or will be carried out in the near future (i.e., results not available yet). It is also slightly unclear whether the study has begun at this stage or not. For example, in the abstract it is stated that the study “is being conducted” whereas in the methods it is stated “ The ChARM programme will be delivered…”

Again, to avoid confusion, the last sentence should be rephrased at the future tense: "the findings will offer ..."

**The description of this study is in the future tense because this is a protocol and indeed, at the time of writing, the study is still ongoing. Thus, the aim of this article is to outline the study methods and activities involved therein. We have now added a sentence in the abstract to make this more clear (p.1, lines 5-6). The text has also been changed to the future tense throughout (i.e. ‘will be conducted’  ‘will offer’ etc.).**

**Introduction**

The introduction is a plea for the prevention of child abuse and neglect. The goal of the program is relevant, as this problem is of major concern for most societies.

The authors make a good demonstration that intervention is less effective when offered once maltreatment is present in the family and that prevention programs should be developed. However, it is not that clear from the methods that they propose a prevention program since targeted families are involved in child protection for child maltreatment (some only at-risk for maltreatment, but some will have documented maltreatment already). The authors also demonstrate that empirically supported programs are useful, but none can prevent child maltreatment on its own since vulnerable families are so hard to reach. Another argument for such a program could be that child maltreatment is explained by multi-systemic factors.

**ChARM is designed to be both an early intervention and prevention programme and for purposes of this study, targeted families will be those deemed to be at risk of child maltreatment as well as those who are currently in contact with social workers due to ongoing child protection concerns. We have now attempted to clarify this point by revising p.3, para 2 accordingly. We have also acknowledged that child maltreatment may be explained by multi-systemic factors (p.2, para 4, line 1).**

Even though we are not questioning the fact that child maltreatment has important consequences on the victims’ development, the study cited to support this link is about child maltreatment prevention in general (Sethi et al., 2013). Perhaps studies examining consequences of child maltreatment (primary or meta-analysis) would be more appropriate to support this link.

**We have amended the text in the Introduction (p.1, final para) to include other studies that have examined the consequences of child maltreatment.**

The authors present the incidence of maltreatment and more specifically of its different forms. In order to do so, they report rates found in meta-analyses of self-reported incidences. Afterward, they provide data for substantiated abuse. It seems to be a good idea to report both since they do not provide exactly the same information. However, when it comes to presenting the rates of the different forms of maltreatment, a little nuancing would be appropriate. In fact, even if it is true that, according to the self-reported rates, neglect is less frequent than physical abuse, this seems to be an under-representation that may be related to the type of measure used.

**We are in agreement with this comment. Therefore, we have added a sentence to the end of para 1 on p.2 to refer to methodological variations across studies.**

Again, in relation to incidence, it is not clear why the authors introduce rates from different countries. When it is written that “prevalence rates of CM are even higher in low and middle-income countries…”, it is unclear why this is relevant to the current study. Does the critique about the unreliable detection and surveillance in most countries apply to Ireland? The pertinence of this discussion is questionable and it is unclear how it reflects the Irish situation.

**Yes, Ireland too has unreliable detection and surveillance systems – we have now indicated this in the first sentence of para 2, p.2.**

The authors mention some protective factors of CM: knowledge of parenting, nurturing parenting skills, parental resilience, social network, etc. These are not protective factors, as protective factors must have made the demonstration they can interrupt or interact with risk factors. These may be viewed as favorable factors or positive factors that MAY prevent CM and promote child well-being.

**In light of this comment, we have re-written this section in order to eliminate any confusion over the terms used.  For example, we have provided a definition of ‘protective factors’ plus further information on p.2, para 4, and p.3, para 1.**

The most important problem with the introduction is that it does not introduce ChARM, but focuses on the WA model of care. We understand that this model is central as it demonstrates the value of an approach that consist of coordinating services around the family. It would be good to discuss strengths and limitations of this model and the need to incorporate empirically-supported programs in WA care. The authors also introduce Meitheal, another program, also inspired by WA. However, there are very few allusions to ChARM, which leads to some confusion. It seems important to discuss the WA model, however I think the authors need to place the focus more on ChARM and to demonstrate the value of this program. Perhaps the progress of the WA implementation in Ireland and Meitheal could be discussed in the discussion?

**We appreciate the above point and we have addressed it by re-locating a paragraph which was originally later in the introduction to earlier (see p. 3, para 2) where the ChARM model is now explicitly mentioned and described.  We have also linked the WA model of care which is introduced in the subsequent para, to the ChARM model (see p.3, para 3, opening sentence), whilst also making more explicit references to ChARM at various junctures thereafter (e.g. p.5,; p.6, para 2). We do believe that the discussion of Meitheal is important here as it provides important background /context for the development of the ChARM model. We have now added subheadings for clarity. Please note also that we have followed the HRB Open guidelines when drafting this article; these require that the detailed description of the intervention be provided within the Method section of the paper.**

**Objectives**

Though the objectives are totally relevant, we do have a concern about a specific hypothesis: (1) the ChARM program will reduce parent-reported incidences of child maltreatment. This comment will be detailed in the method, but the administrative data is considered as a secondary outcome, which is not consistent with the introduction.

**We have responded to this comment in sections below.**

**Methods**

Since we are now in 2018, and the recruitment was planned for 2015-2017, would it be possible to have an update and the authors to state the exact number of participants they recruited? The authors could then perform post hoc computations of statistical power.

**We appreciate why the reviewers would like an update on the study, but given that this is a protocol, we do not believe that it would be appropriate to provide an update on the study within the body of the manuscript. Data collection has just been completed and we have indicated this under the heading ‘trial status’ on p.15.**

There is slight inconsistency in the targeted age of the children. Sometimes it is written 3-10 and sometimes it is 3-11. Please verify.

**We apologise for this oversight – this has been amended to the correct age band throughout (3-11 years old) highlighted sections on pp.1, 3, 6, 7, 15, 31 and 32).**

In this section, it is said that the ChARM program prevents child maltreatment “within intact families”. We were not sure if the term “intact family” refers to families in which both biological parents are living together or family in which the child is not in out-of-home placement.

**The ChARM intervention is evaluating a wraparound-inspired approach to the prevention of child maltreatment with families of children still living in the home. Therefore, we have amended the text accordingly (p.1, para 1; p.3, paras 2 and 4).**

The objectives of the study are relevant, clear and precise. However, the first hypothesis is not clear. Why should the parent-reported incidences of child maltreatment decrease? Is it that parents will report having less maltreating behavior after the program?

**Yes, we believe that, if the programme is found to be effective, it should help to reduce maltreating behaviour in parents, although we are also aware that this is a difficult outcome to measure due to the need to rely solely on parent report.**

The introduction convinced us that the program to be evaluated is a prevention program of maltreatment, implemented in the community with families that are at risk to commit child maltreatment. We were surprised to see that participants were actually followed by child welfare, including some that had already committed child maltreatment. This apparent inconsistency may be due to differences between child protective services in Ireland compared to our system in Quebec (if it is the case, a brief description of CPS in Ireland could help). Or maybe ChARM is not a prevention program but an intervention one.

**ChARM has been designed as an early intervention and prevention programme for children still living within the home. This means that whilst it is a prevention programme first and foremost, it may also be used as a form of early intervention for ‘at risk’ families in which there are early signs of child maltreatment ,but where these are not sufficiently severe to warrant placement (as yet) in state care (please see second inclusion criterion on p. 7). As described on p.7 (para 1), social care professionals in Ireland use the Hardiker model to determine the level of risk in the families with whom they work (see Figure 2). From anecdotal evidence, we know that these professionals are reluctant to remove children from their families unless absolutely necessary.**

**A brief description of Child Protective Services in Ireland is now provided, as requested, on p.5, para 1 (with a new sub-heading also added).**

The first and third exclusion criteria seem to be redundant with inclusion criteria.

**We understand why this might be perceived as such, but we felt it was necessary to include exclusion criteria points 1 and 3 for purposes of total clarity.**

The eligibility criteria for program providers to be part of the study is not precise enough. What do you mean by “considerable experience”? In what capacity are they working within CPS? How much experience is required in order to deliver the components? It could be interesting to quantify this experience in months/years.

**We have revised and added the additional text on the eligibility criteria for program providers on p.7 (penultimate para).**

***The most important problem with this protocol is the number of participants to the study. 50 (25+25) over two years, seem to be very few, especially after the authors mention that this is a prevalent problem. If probing practices are implemented in order to reach and engage families, how do you explain such a small n? An analysis of the statistical power is essential. Indeed, we have to know if the number of participants available in your study and the number of outcomes to consider make it possible to reach your goals. It would be disappointing for everyone that you come to the conclusion that you cannot state on the effectiveness of ChARM since your negative results are due to insufficient statistical power.

This will probably not be enough to test moderations. You state that 150 families would be necessary in order to detect a 0,8 effect size, which is quite big for this population and this context. Are you willing to accept that with 50, you wouldn’t detect a 0.8 effect size? I read that the authors are aware of this problem. But we think they really need to find solutions.

**Yes this is a problem in many ‘real world’ trials of this nature. We have encountered many difficulties and challenges beyond our control in the design and development of this study. Most notably, a major restructuring of services and staff within the Tusla Child and Family Agency (with whom we are working) took place at the same time as the study was being designed and we met with subsequent difficulties in securing collaborating sites. This hugely affected our projected recruitment and proposed sample size, with the result that the sample is much smaller than originally anticipated (or calculated).  This small sample size also reflects the challenges inherent more generally in recruiting families with high needs to research programmes (Horowitz et al. 2002).  Furthermore, we realise that 150 families is, by any standards, very large and it is debatable as to whether or not we would have achieved that number, had everything gone according  to plan in terms of our collaborating sites and projected recruitment**. 

**Having said that, we are reluctant to abandon the trial at this point and it has become a more exploratory piece. At a minimum, we will obtain very useful information on these vulnerable families in terms of their profile and outcomes over time. Furthermore, we have an embedded process evaluation which, we believe will yield very interesting results on the implementation of these kinds of programmes, barriers/challenges to implementation and how they might be improved.**

Since the protocol is so complete, we are interested to know more about the way contamination will be measured and controlled statistically if needed.

**We are unsure if the reviewers are referring to contamination of the research team to treatment condition (i.e. unblinding) or contamination of the control group (i.e. where the control group receive some or all of the intervention).  Regarding the former, issues relating to potential unmasking are described in the randomisation and blinding section (p.9). Potential contamination of the control group is also addressed on p.9, para 2 (‘Contamination’ Section). As noted in the manuscript, to reduce the threat of contamination, practitioners delivering the intervention will not be involved in delivering usual services to control group participants. After intervention delivery, we will also ask practitioners to report whether any contamination of the control group occurred. Relevant statistical controls are also described (see p.9).**

**In addition, the Service Utilisation Questionnaire will provide information on if/and how contamination has occurred by eliciting information on the various ‘in-house’ and other services used/accessed by parents since the date of entry into the study.**

Since the age of the children show a wide range, what program is used from IY? The preschool or the school age basic program?

**The Incredible Years school age Basic Programme will be used. This is delivered in two-hour, weekly group sessions over 14 weeks.**

We would like to know more about the name and number of "subscales" for each measure used. Could you also give some information on the psychometric value of the selected tools? I also notice that these are several measures for very few participants. What correction are you planning to use to compensate, knowing that Bonferroni is relatively conservative... In addition, I invite the researchers to question the potential impact of the number of measures on the recruitment of participants. Indeed, the number of questionnaires provided in the protocol could have an impact on the possibility of generalizing the results to all the very vulnerable parents who would have refused to participate in the research.

**Furthers details on the scales and subscales for each measure that will be used in this study are now provided in the supplementary material (p.25). We have also added a new para on p.11 (para 2) to address this point.**

**We acknowledge that the number of measures which participants are required to complete may be time-consuming and there is the potential that this may deter vulnerable parents to refuse to participate in the research. However, we have used multiple test batteries in our previous (and current) work with vulnerable parents and children and this has not affected our recruitment (or retention) rates which have remained strong. We are acutely aware of the issue of participant burden and we have endeavoured to address this in detail as part of our ethical approaches and procedures as outlined in the ethics application pertaining to this study (which was approved by the Maynoooth University Social Research Ethics Sub-Committee. For instance, care will be taken to minimise participant burden where possible and it is estimated that individual data collection sessions will take approximately 40-60 minutes, although this is likely to vary across participants depending on their level of literacy. Breaks will also be provided as and when required and an additional visit made at a later date if necessary, in order to complete data collection. It has been our experience that families (both vulnerable and otherwise) enjoy meeting with a researcher and that, at times, there may also be therapeutic benefits for the families concerned (McGilloway et al. 2012).**

**We appreciate the potential statistical challenges of using multiple outcome measures, but we are also aware that this is not straightforward and also quite a controversial issue. Firstly, we have selected only two primary outcomes against which we will test our main hypotheses. Secondly, with regard to the other findings, we will follow the guidelines suggested by Feise (2002). Thus, we will evaluate the quality of study and the amplitude (effect size) of all findings before interpreting their statistical significance whilst also referring to other studies in the literature. In addition, we will regard the findings as tentative until they are corroborated.  According to Feise, “a single study is most often not conclusive, no matter how statistically significant its findings”. This is particularly so here in view of the exploratory nature of the study.**

The WAI is used to measure the capacity to engage parents. This is known to be a measure of working alliance. There is at least two versions of this questionnaire: parent and therapist. Why not use the parent version? Furthermore, when is it going to be completed? Some researchers have shown that the validity of this measure and its capacity to detect relevant working alliance is at its best around the third meeting. We invite the authors to have a discussion about this question of timing.

**We will be administering both versions of the WAI in the process evaluation of ChARM – the practitioner and parent versions – in order to assess practitioner –parent relationships. Table 2 (p.31) has been amended accordingly in order to avoid any confusion. We appreciate the point in relation to the optimal validity of the measure at the third meeting; in this case, the WAI will be completed upon completion of the IY programme at which stage, both the parents and practitioners will have established and built up a relationship, having been in contact for 18 weeks in total (4 weeks PLSP, 14 IYPP). This should allow us to detect a meaningful working alliance.**

We wonder why you presented maltreatment report by parents as a primary outcome, and CPS report of maltreatment as a secondary outcome. We believe that because the parents are followed in CPS, which is a pretty coercive context, they could be tempted to give answers to their advantage which can cause a social desirability bias to this outcome. That is why we also suggest the use the official CPS report of maltreatment in combination to the parent-reported maltreatment as primary outcomes.

**Ideally, we would have preferred to have used CPS reports of maltreatment as our primary outcome and indeed, we explored this in our initial discussions with the service providers. However, we understand that this information is not routinely recorded in Ireland, nor is it always accurate. There were attendant concerns around confidentiality/accessibility by the research team. For this reason, we selected parent-reported incidences of child maltreatment (assessed using he Conflict Tactics Scales Parent-Child – Short Form Amended (CTSPC – SFA) (Straus et al. 1998), as our primary outcome alongside child behaviour and wellbeing as the second primary outcome (assessed using both the parent- and child-report versions of the Strengths and Difficulties Questionnaire (SDQ) (Goodman 1997)).**

****

**However, we are aware that there is likely to be some degree of socially desirable responding by parents on the former and indeed, this has also been reported in other research (Hurlburt et al 2013).  Therefore, we hope to address this, at least indirectly, through interviews with the practitioners who are working with the families and who have access to their records. At a minimum, we hope to be able to ascertain the existence of any out-of-home placements that may have occurred during the study (as a secondary outcome).**

It is a very interesting idea to have interviews with those who abandon the program. I wonder how they will be recruited. If they do not agree to go through the program, it is not very likely that they will accept the go further with the research process. In addition, those who could accept would have specific characteristics to be considered.

**We acknowledge that parents who have dropped out of the programme may refuse to subsequently speak to a researcher, but we have used this approach successfully in our previous research (e.g. Furlong and McGilloway, 2014). All parents will be asked to provide their written informed consent to agree to be contacted by the research team irrespective of whether or not they complete the ChARM programme. We also provide a small shopping voucher as a ‘thank you’ to all participants for taking part in interviews/assessments.  With regard to the characteristics of ‘leavers’  who are willing to be involved in research, in our previous work, we recruited ‘drop-out’ parents purposively  using a range of criteria , such as age, marital status, age of the child, location etc. This enabled us to recruit a heterogeneous sub-sample and to sidestep, for the most part, the issue of response bias.**

We are also very satisfied that the fidelity of implementation of the program that is described in this study. However, we question the validity of using it as a moderator, because to be eligible as a moderator, a variable should respect criteria of temporal precedence and independence with the program (Kraemer, Kiernan, Essex & Kupfer, 2008).

**We understand that it is desirable for moderator variables to be uncorrelated with both the predictor and criterion variables, but here, we believe that the ‘site readiness’ aspect of programme fidelity is an important situational factor/element of the environment or context in which the model will be delivered; thus, it are may legitimately influence the direction and strength of the intervention effect. However, participant engagement (dosage) ought not to have been included here as a moderator, because it is a mediator, hence the confusion. Therefore, we have amended accordingly the relevant section of on p.13, para 3. In addition, we should point out that moderator effects will only be explored if they meet the criteria outlined by Kraemer et al. (2002) and will be carefully chosen given the limited sample size.**

Thus, many good ideas, but perhaps not the statistical power to accomplish all this!

**We share the reviewers’ concerns in relation to the small sample size and a number of other challenges, but we believe that this research is worth pursuing and we will, of course, take care to acknowledge the exploratory nature of the study and its limitations at the time of reporting the findings.**

**Discussion**

Very short. More information regarding the anticipated limitations of the study would be helpful.

**As outlined earlier, the research team have followed the guidelines of HRB Open Access in preparing this article and in so doing, the paper adheres to the SPIRIT reporting guidelines which do not stipulate a specific limitations section.  However, we have now included some brief information on limitations of the study on p.15, para 2.**

**Ethics Standards**

We appreciated the section about how the authors are planning on complying with ethical standards. This section, like all others, is precise and detailed. It shows the authors’ concern to follow best practices in program evaluation with vulnerable clientele.

**General**

There are some language errors or typos. In the second paragraph, it is written that “In Ireland, over 40,000 referrals of child welfare and abuse cases.” I suppose the authors mean abuse and neglect cases?...

**No, this is not a typo. We are referring to both child welfare concerns and abuse cases here because, in Ireland, data are collected on both together.**

On p.5, paragraph starting with “This study involves the evaluation of…” and in the next one starting with “The objectives of the study…”, there are many typo problems. Many little “me” are inserted, and I cannot explain why. See the whole text for other problems of this kind.

**All of these typos have now been corrected (e.g. on p.6 as highlighted and throughout paper (removal of ‘me’) (p5., para 4; p.6, paras 3, 4,).**
